# Prevalence of diagnosed temporomandibular disorders: A cross-sectional study in Brazilian adolescents

**DOI:** 10.1371/journal.pone.0192254

**Published:** 2018-02-08

**Authors:** Fernanda Mara de Paiva Bertoli, Carolina Dea Bruzamolin, Eduardo Pizzatto, Estela Maris Losso, João Armando Brancher, Juliana Feltrin de Souza

**Affiliations:** 1 Graduate Program in Dentistry, Positivo University, Curitiba, Paraná, Brazil; 2 Department of Stomatology, School of Dentistry, Universidade Federal do Paraná, Curitiba, Paraná, Brazil; University of Washington, UNITED STATES

## Abstract

**Background:**

The prevalence of signs and symptoms of temporomandibular disorders (TMD) increases during adolescence and adulthood. Few studies have examined TMD prevalence in Brazilian adolescents.

**Aim:**

To investigate the prevalence of TMD in Brazilian adolescents.

**Methods:**

A representative population-based sample of 934 adolescents (10–14-years-old) was examined. TMD screening was performed using a questionnaire by the American Academy of Orofacial Pain. TMD diagnoses used research diagnostic criteria for temporomandibular disorders (RDC/TMD—Axis-I). Examinations were performed by a single calibrated examiner (kappa > 0.80).

**Results:**

The prevalence of TMD symptoms was 34.9%; the most frequently reported symptoms were headache and neck ache (20.9%), followed by joint sounds (18.5%). Myofascial pain was the most prevalent type (10.3%), followed by disc displacement with reduction (8.0%) and arthralgia (3.5%). There was a significant association between sex and TMD symptoms; prevalence was significantly higher in girls (RP = 1.37; 95% CI = 1.14–1.65; p = 0.001). Myofascial pain of TMD and displacement with reduction were more prevalent in girls (RP = 1.76; p = 0.007 and RP = 2.06; p = 0.004, respectively).

**Conclusion:**

TMD symptoms were present in 34.9% of adolescents, with myofascial pain being the most prevalent type (10.3%). TMD was significantly more common in girls. Routine pediatric dental care should include a TMD screening.

## Introduction

Temporomandibular disorders (TMD) are a set of joint and muscular dysfunctions of the cranio-orofacial area. They are primarily characterized by joint and/or muscular pain, noise in the temporomandibular joints (TMJ), and limited or irregular mandibular function. TMD can considerably affect quality of life [1]. The American Academy of Pediatric Dentistry recognizes that disorders of the TMJ, masticatory muscles, and associated structures occasionally occur during childhood and adolescence, but with less intensity than in adult populations. During adolescence, TMJ presents with mild or moderate signs and symptoms [2].

The reported prevalence of TMD in infants, children, and adolescents varies widely in the literature, due to differences in the populations studied, diagnostic criteria, examination methods, and inter- and/or intra-rated variations of examining practitioners [3]. Furthermore, studies have demonstrated that the prevalence of TMD symptoms and signs is rare in early childhood [4]. However, it becomes more prevalent in adolescence [5] and adulthood, with a prevalence ranging from 4.9% [6] to 60% [7], with the incidence of signs and symptoms generally increasing with age [8]. Most adults affected by TMD report that symptoms originally developed during adolescence [9].

Therefore, a differential diagnosis between the growth process of the structures and the disorders is necessary for early prevention. In addition, it remains unclear whether these signs and symptoms represent a deviation from normality, are preclinical findings of a disease state [10], or are part of adaptive physiological changes of the craniofacial complex [11]. Moreover, TMD can clinically present in several aspects, varying in terms of the structure involved as well as the severity of the symptoms and signs, and these may be even more difficult to recognize during childhood.

The diagnosis of early symptoms and signs of TMD during adolescence is fundamental for preventing or minimizing TMD pain and for reducing its impact on adolescents’ lives [12]; however, little is known about TMD in Brazilian adolescents. Thus, the aim of this study was to investigate the prevalence of TMD in adolescents according to the research diagnostic criteria for temporomandibular disease (RDC/TMD).

## Material and methods

This study received approval from the Research Ethics Committee of Universidade Positivo (Process no. 879.404). This cross-sectional study included adolescents aged 10–14 years, attending public and private schools in the city of Curitiba, Paraná, Brazil. The city has approximately 1.893.997 inhabitants and has a Human Development Index of 0.823. The schools are geographically distributed in nine sanitary districts. The parent or legal guardian responsible of the participants received written and verbal information about the study, and signed consent was obtained from a parent for all participants. The study adhered to the tenets of the Declaration of Helsinki.

### Study population

The sample size calculation considered 5% accuracy, a confidence interval (CI) of 95%, a previous TMD prevalence of approximately 25.5% in adolescents [13], and the population of adolescents of the target age in the city of Curitiba. According to the Department of Municipal and State Education, approximately 65.074 children were of the correct age; 76.4% attended public schools and 23.6% attended private schools. Considering the effect of stratified sampling (1.5), a representative sample should be 800 adolescents.

For a stratified representative probabilistic population-based sample, a random sampling procedure was applied to select a proportional sample according to sanitary district and teaching systems. A total of 19 schools, two per sanitary district, were randomly selected. The school randomization was performed using a website (http://www.randomizer.org).

The exclusion criteria were individuals with pain of odontogenic origin, with orthodontic appliances, occlusal splints, dental prostheses, severe facial or dental anomalies, extensive dental destruction, systemic disorders with cognitive or behavioral problems, and speech disorders. Those using medications, such as antidepressants, muscle relaxants, and nonsteroidal anti-inflammatory drugs; or adolescents who declined to participate, were also excluded.

### Data collection

Previously, a pilot study was performed at a private school in Curitiba, which was not considered part of the study sample. The questionnaire was pre-tested in this sample; it was considered adequate for the study population and no alterations were made. One examiner (F.M.P.B.) with more than 10 years of practice was calibrated for applying clinical RDC/TMD criteria, and twice examined a sample of 30 adolescents aged 10–14 years, with an interval of 15 days.

The intraexaminer coefficient of concordance (kappa) for TMD according to the RDC/TMD was >0.80. In addition, questions about general health and, for adolescent girls, about menarche were applied. The data were collected from September 2014 to July 2016.

### TMD screening

For TMD screening, the presence of self-reported symptoms of TMD was determined using a valid Portuguese version of the self-reported questionnaire by the American Academy of Orofacial Pain (AAOP) [14]. This questionnaire had previously been validated by Franco et al. [14] as showing good reliability and validity for screening for TMD in Brazilian adolescents. It is composed of 10 dichotomous questions about TMD symptoms and was used to screen adolescents with TMD symptoms [13,15]. All participants who met the inclusion criteria (n = 934) answered the questionnaire, and adolescents that had at least one positive answer were clinically examined (n = 345).

### Clinical examination

The clinical examinations were carried out in the school environment. The student and the researcher remained seated in chairs in front of a window in order to obtain the maximum natural lighting. For TMD characterization, some findings of the examination protocol of the Portuguese version of the RDC/TMD Axis I were used. According to the literature, the Axis I instrument can be applied to both adults and adolescents [16]. However, to prevent false positive results in children, ambiguous, uncomfortable, and prolonged procedures, such as intra-auricular and intra-oral palpation, are excluded [8].

On the basis of the RDC/TMD Axis I summary of findings, the participants were categorized into one of three RDC/TMD groups. Group I included muscle diagnosis (myofascial pain and myofascial pain with limited opening). Group II included disc displacements (with and without reduction), and Group III included arthralgia, osteoarthrosis, and osteoarthritis. Axis II was not used, because it has not been validated for use with children and adolescents younger than 13 years [17].

### Data analysis

Data were analyzed using SPSS Statistics for Windows, Version 20.0 (IBM Corp., Armonk, NY, USA) and Stata (StataCorp LLC, College Station, TX, USA). The dependent variable was TMD, which was categorized as the presence or absence of TMD symptoms according to the AAOP questionnaire.

The associations between independent variables and types of TMD were analyzed using the χ^*2*^ test. The crude prevalence ratio (PR_c_) and 95% CI were calculated by Poison regression analysis. A p value < 0.05 was considered significant.

## Results

A total of 4,055 adolescents were invited to participate in the present study. Of those, 934 adolescents returned the signed consent form, met the inclusion criteria, agreed to participate, and were present when the evaluations were performed (a response rate of 23.1%) (S1 File). Of the participants, 55.4% (n = 518) were girls; 36.1% (n = 187) of them had experienced menarche. The mean age of the participants was 11 years, and most attended public school (87.7%, n = 820) (Table 1).

**Table 1:** Demographic characteristics of the study population (Curitiba, Paraná, Brazil, 2016).

Age (yr)	n (%)	Total
10	260 (27.8)	
11	321 (34.3)	
12	207 (22.1)	
13	87 (9.3)	
14	59 (6.3)	
Mean age*	11.32 (1.2)	934
**Gender**		934
Female	518 (55.4)	
Male	416 (44.5)	
**Menarche**		517
Yes	187 (36.1)	
No	330 (63.8)	
**School type**		934
Public	820 (87.7)	
Private	114 (12.2)	

*Numbers are given as mean and standard deviation (SD).

The data from self-reported TMD (i.e., TMD symptoms) are shown in Table 2 (S1 Table). The prevalence of TMD symptoms was 34.9%, with 206 (39.8%) girls and 120 (28.8%) boys reporting symptoms. The most reported symptoms were headache and neck ache (20.9%), followed by joint noises (18.5%) and pain around the TMJ (14%).

**Table 2:** Prevalence of symptoms of TMD according to the AAOP questionnaire (Curitiba, Paraná, Brazil, 2016).

Related Symptoms	Yes n (%)		Total Yes n (%)
	Girls	Boys	
TMD prevalence—AAOP questionnaire	206 (39.8)	120 (28.8)	326 (34.9)
Do you have difficulty, pain, or both when opening your mouth, for instance, when yawning?	33 (6.4)	13 (3.1)	46 (5.0)
Does your jaw “get stuck”, “locked”, or “go out”?	19 (3.7)	7 (1.7)	26 (2.8)
Do you have difficulty, pain, or both when chewing, talking, or using your jaws?	35 (6.8)	12 (2.9)	47 (5.1)
**Are you aware of noises in the jaw joints?**	108 (20.8)	65 (15.6)	173 (18.6)
Do you have pain in or near the ears, temples, or cheeks?	97 (18.7)	34 (8.2)	131 (14.1)
**Do you have frequent headaches or neck aches?**	139 (26.8)	56 (13.5)	195 (20.9)
Do you have frequent toothaches?	48 (9.3)	26 (6.3)	74 (8.0)
Have you had a recent injury in your head, neck, or jaws?	29 (5.6)	26 (6.3)	55 (6.1)
Have you been aware of any recent changes in your bite?	25 (4.8)	24 (5.8)	49 (5.9)
Have you been previously treated for unexplained facial pain or a jaw joint problem?	8 (1.5)	11 (2.6)	19 (97.8)

AAOP, American Academy of Orofacial Pain; TMD, temporomandibular disorder.

The prevalence of TMD diagnosis in adolescents is presented in Table 3. Myofascial pain was the most prevalent disorder (10.3%), followed by disc displacement with reduction (8.0%), arthralgia (3.5%), and myofascial pain with opening limitations (0.2%). No cases of disc displacement without reduction, osteoarthritis, or osteoarthrosis were detected.

**Table 3:** Prevalence of TMD diagnoses according to the RDC/TMD (n = 934). (Curitiba, Paraná, Brazil, 2016).

TMD Subtypes	Yes n (%)		Total Yes n (%)
	Girls	Boys	
I.a Myofascial pain with limited opening	2 (0.4)	0	2 (0.2)
I.b Myofascial pain	66 (12.7)	30 (7.2)	96 (10.3)
II.a Disc displacement with reduction	54 (10.4)	21 (5.0)	75 (8.0)
II.b Disc displacement without reduction with limited opening	0	0	0
II.c Disc displacement without reduction without limited opening	0	0	0
II. a Arthralgia	21 (4.1)	12 (2.9)	33 (3.5)
III.b Osteoarthritis	0	0	0
III.c Osteoarthrosis	0	0	0

RDC/TMD, research diagnostic criteria for temporomandibular disease; TMD, temporomandibular disorder.

There was a significant association between sex and TMD symptoms; girls had a significantly higher prevalence of symptoms than boys (RP = 1.37; 95% CI = 1.14–1.65; p = 0.001) (Table 4). Moreover, girls also had a significantly higher prevalence of myofascial pain (RP = 1.76; 95% CI = 1.17–2.66; p = 0.007) and disc displacement with reduction (RP = 2.06; 95% CI = 1.26–3.36; p = 0.004) (Table 5; Table 6).

**Table 4:** Relationship between prevalence of TMD symptoms and age, sex, menarche, and school (Curitiba, Paraná, Brazil, 2016).

Characteristics	TMD [n (%)]			Prevalence Ratio (95% CI)	p value
	Yes	No	Total		
			n		
**Age (y)**					
10	86 (33.1)	174 (66.9)	260	1.00	-
11	112 (34.9)	209 (65.1)	321	1.05(0.83–1.32)	0.647
12	69 (33.3)	138 (66.7)	207	1.00(0.77–1.30)	0.953
13	38 (43.7)	49 (56.3)	87	1.32(0.98–1.77)	0.065
14	21 (35.6)	38 (64.4)	59	1.07 (0.73–1.58)	0.709
**Sex**					
Female	206 (39.7)	312 (65.1)	518	1.37 (1.14–1.65) 1.00	**0.001**
Male	120 (28.8)	296 (71.2)	416		
**Menarche**					
Yes	75 (40.1)	112 (59.9)	187	1.01 (0.81–1.25)	0.927
No	135 (40.9)	195 (59.1)	330	1.00	
**School**					
Private	48 (42.1)	66 (57.9)	114	1.00 0.80 (0.63–1.01)	0.071
Public	278 (33.9)	542 (66.1)	820		

CI, confidence interval; TMD, temporomandibular disorder

A p value < 0.05 is considered significant, as determined by the Poisson regression analysis.

**Table 5:** Relationship between prevalence of myofascial pain and age, sex, menarche, and school (Curitiba, Paraná, Brazil, 2016).

Characteristics	Myofascial [n (%)]			Prevalence Ratio (95% CI)	p value
	Yes	No	Total		
**Age (y)**					
10	24 (9.2)	236 (90.8)	260	1.00	
11	31 (9.7)	290 (90.3)	321	1.04 (0.62–1.73)	0.861
12	17 (8.2)	190 (91.8)	207	0.88 (0.49–1.61)	0.700
13	13 (14.9)	74 (85.1)	87	1.61 (0.86–3.03)	0.134
14	11 (18.6)	48 (81.4)	59	2.01 (1.04–3.89)	0.036
**Sex**					
Female	66 (12.7)	452 (87.3)	518	1.76 (1.17–2.66) 1.00	**0.007**
Male	30 (7.21)	386 (92.79)	416		
**Menarche**					
Yes	27 (14.4)	160 (85.6)	187	1.22 (0.77–1.92) 1.00	0.391
No	39 (11.8)	291 (88.1)	330		
**School**					
Private	9 (7.9)	105 (92.1)	114	1.00 1.34 (0.69–2.59)	0.379
Public	87 (10.3)	733 (89.4)	820		

CI, confidence interval; TMD, temporomandibular disorder

A p value < 0.05 is considered significant, as determined by Poisson regression analysis. Significant values are shown in bold.

**Table 6:** Relationship between prevalence of disc displacement and age, sex, menarche, and school (Curitiba, Paraná, Brazil, 2016).

Characteristics	Disc Displacement with Reduction [n (%)]			Prevalence Ratio (95% CI)	p value
	Yes	No	Total		
**Age (y)**					
10	17 (6.5)	243 (93.5)	260	1.00	**-**
11	29 (9.1)	292 (90.9)	321	1.38 (0.77–2.45)	0.271
12	15 (7.3)	192 (92.7)	207	1.10 (0.56–2.16)	0.764
13	9 (10.3)	78 (89.7)	87	1.58 (0.73–3.42)	0.244
14	5 (8.5)	54 (91.5)	59	1.29 (0.49–3.37)	0.595
**Sex**					
Female	54 (10.4)	464 (89.6)	518	2.06 (1.26–3.36)	**0.004**
Male	21 (5.1)	395 (94.9)	416	1.00	
**Menarche**					
Yes	17 (9.1)	170 (90.9)	187	0.81 (0.46–1.39)	0.451
No	37 (11.2)	293 (88.8)	330	1	
**School**					
Private	7 (6.1)	107 (93.9)	114	1	0.434
Public	68 (8.0)	752 (92.0)	820	1.35 (0.63–2.86)	

CI, confidence interval; TMD, temporomandibular disorder.

A p value < 0.05 is considered significant, as determined by the Poisson regression analysis.

Myofascial pain with limited opening and arthralgia were observed in few cases and showed no relationship to the independent variables.

## Discussion

The diagnosis of TMD is a challenge in pediatric dentistry. The early signs of TMD should be discerned from other adaptive physiological changes during growth of the craniofacial complex. Thus, standard clinical criteria for TMJ must be applied to improve the validity of the results.

Previous studies have used the RDC/TMD in children and adolescents, with good reliability [14,13,17]. These diagnostic criteria have been used as a gold standard for both children and adults, improving clinical TMD examination accuracy and reproducibility. Recently, another version of the RDC/TMD was published, i.e., the diagnostic criteria for TMD (DC/TMD) [18]. However, these have not been validated for use in children [1] and are not available in Portuguese. Moreover, the conventional RDC/TMD criteria comprise the most acceptable and well-known standard for diagnosing TMD in research [14]. The majority of epidemiological studies have described the prevalence of TMD in adults; few studies use RDC/TMD classification in children or adolescents. Studies of younger patients have not diagnosed TMD, otherwise they have defined the presence of one or more signs and symptoms [8].

In the present study, a total of 206 (22.0%) adolescents were diagnosed positively for any TMD subtype (overall TMD) by RDC/TMD Axis I. The prevalence reported here is lower than those reported in other studies using RDC/TMD: 25.5% [12,19]; 27.2% [1], and 30.4% [13]. The higher prevalence in these studies could be explained by the higher age of the participants.

Regarding self-reported symptoms results, the prevalence of reported symptoms was 34.9%. Our results show that the adapted Portuguese version of the AAOP questionnaire had good reliability and validity for screening TMD in Brazilian adolescents [14] once difficulties of comprehension had been resolved. In this population, the prevalence of TMD signs and symptoms were lower than those reported by Santis et al. [15] using the same criteria. The authors observed that the prevalence of self-reported signs and symptoms were, respectively, 54.55% and 20% [15]. The questionnaire is useful for pre-screening of patients, but does not facilitate a definitive diagnosis [15], as the results have high sensitivity but low specificity.

Myofascial pain was the most prevalent TMD symptom (10.3%), followed by disc displacement with reduction (8.0%), in agreement with some studies that reported myofascial pain as the most prevalent diagnosis [13,20]. No case of disc displacement without reduction, osteoarthrosis, or osteoarthrosis was diagnosed. These results were corroborated by other authors; these symptoms are uncommon among children and adolescents [1,13].

Many studies have reported that symptoms of masticatory system disorders are more frequent in females than in males [5,9,21,22], possibly due to biological differences, including hormonal and psychosocial factors [23,22]. In the present study, girls showed a higher prevalence of TMD symptoms, myofascial pain, and disc displacement with reduction. Our results corroborated those of Tecco et al. [21], who investigated the signs and symptoms of TMD in 1,134 subjects (593 males and 541 females) ranging from 5 to 15 years of age. However, other studies of this age range did not find differences between the sexes [1,12,16], probably because the difference in signs and symptoms between sexes was small in childhood. From late adolescence, girls reported more symptoms and exhibited more clinical signs than boys [10].

No difference in the prevalence of TMD was found in girls before and after menarche, in accordance with previous studies [12,16]. LeResche et al. [24], who studied pubertal stages and TMD pain, observed an increase in TMD pain in adolescents with advancing of pubertal stage. According to the authors, girls until 11-years-old were in pre-pubertal stage and after 12 years of age, most girls were in pubertal/post-pubertal stage. However, according Rapkin et al. [25], the age may be the less important characteristics to determine the pubertal stage than clinical and research settings, such as the hormonal markers. Thus, an important limitation of the present study is that the pubertal stages were not completely evaluated. Menarche was the only sign of pubertal stage evaluated. Considering our sample, the most girls had not yet manifested menarche, a small sample of girls had manifested menarche, so might not have been sufficient to show a statistical difference.

Although the sample was representative of the city, the present study was limited in that the sample was inhomogeneous in terms of age; there were few older adolescents (13 and 14 years of age). This may be due to the high rate of non-participation among older adolescents. This limitation may also explain the absence of differences in the prevalence of TMD in girls before and after menarche. Previous studies have shown that headaches are commonly associated with TMD signs in children [26]. Headache was the most-reported symptom of TMD; it was reported by 20.9% of subjects, and these findings are in accordance with the results of other studies (21.65% [11] and 20% [27]). Our results are based on self-reporting of a single question; thus, the diagnosis of headache was not classified. In a previous study, headaches were evaluated in children and adolescents, and signs and symptoms of TMD were observed in significantly more patients with headaches than in the control group [28].

In another study of Brazilian adolescents, the most prevalent symptoms were TMJ sounds (26.72%) [11]. We found that 18.5% of study subjects self-reported articular sounds, however, another study found a high prevalence of joint noises (82.4%) [29]. It is known that TMD signs and symptoms in adolescence are often occasional and fluctuating, especially clicking sounds [16,30], which usually increase with age. Another causal factor for articular sounds may be a transitory incompatibility of the disc contour relative to the fossa and condyle contour originating from differential growth rates and calcification [31].

In the present study, crepitation was detected in less than 1% of subjects; this is a rare finding in children [8,2,30]. In a longitudinal study of children 5 years of age, Torii et al. [32] related that clicking was observed in 48% of subjects; however, this symptom was temporary in many subjects, and only 5% had persistent clicking. Persistent clicking is thought to be a more important symptom than temporary clicking, as persistent clicking or crepitation can cause TMJ locking, resulting in the need for treatment.

The MVO results of this study are similar to previously reported findings [31]. The clinical range of mandibular motion of 12- to 18-year-olds is not significantly lower than that of adults, and the cut-off points in the motion range in RDC/TMD have not changed [17].

Considering the signs and symptoms of TMD can appear at an early age, routine dental examination should include an evaluation to identify patients who should be observed more closely [10]. The alterations in TMJ usually begin at very young ages, and hence, it is important to identify those elements of the temporomandibular joints that may lead to development of disorders, as the continual process of growth that these structures undergo in children affords great potential for biological adaptation, which in an adult can trigger a functional alteration or pathological process [26]. Important differences between children and adults must be considered in the diagnosis of TMD, as biological structures are undergoing differential patterns of growth and development and cognitive development and comprehension during development.

In conclusion, in the present study, TMD symptoms were present in 34.9% of adolescents, with myofascial pain being the most prevalent type (10.3%). TMD was significantly more common in girls, but menarche was not associated with the prevalence of TMD in this sample. Considering that signs and symptoms can increase with age, clinicians and pediatric dentists should be prepared to diagnose early TMD signs for management of this condition.

## Supporting information

S1 File Data set from the study. (XLSX)

S1 Table Questionnaire and prevalence of symptoms of TMD. (DOC)
