# Supplementary material for: Prevalence of diagnosed temporomandibular disorders: A cross-sectional study in Brazilian adolescents
Source: PLoS One. 2018 Feb 8;13(2):e0192254. doi: 10.1371/journal.pone.0192254 (PMC5805263; doi:10.1371/journal.pone.0192254)
Supplement: S1 Table — (DOC) [file pone.0192254.s002.doc]

**S1A Table. Questionnaire for detection TMJ symptoms according American Association of Orofacial Pain.**

1. Do you have difficulty, pain, or both when opening your mouth, for instance, when yawning?
2. Does your jaw “get stuck”, “locked”, or “go out”?
3. Do you have difficulty, pain, or both when chewing, talking, or using your jaws?
4. Are you aware of noises in the jaw joints?
5. Do you have pain in or near the ears, temples, or cheeks?
6. Do you have frequent headaches or neck aches?
7. Do you have frequent toothaches?
8. Have you had a recent injury in your head, neck, or jaws?
9. Have you been aware of any recent changes in your bite?
10. Have you been previously treated for unexplained facial pain or a jaw joint problem?

**S1B Table.** **Prevalence of symptoms of TMD according to the AAOP questionnaire.**

| **Related Symptoms** | **n (%)** | | **Total** |
| --- | --- | --- | --- |
|  | **Yes** | **No** |  |
| TMD prevalence - AAOP questionnaire | 326 (34.9) | 608(65.1) | 934 |
| Do you have difficulty, pain, or both when opening your mouth, for instance, when yawning? | 46 (4.9) | 888 (95.0) | 934 |
| Does your jaw “get stuck”, “locked”, or “go out”? | 26 (2.8) | 908 (97.2) | 934 |
| Do you have difficulty, pain, or both when chewing, talking, or using your jaws? | 47 (5.1) | 887 (94.9) | 934 |
| **Are you aware of noises in the jaw joints?** | 173 (18.5) | 761 (81.4) | 934 |
| Do you have pain in or near the ears, temples, or cheeks? | 131 (14.0) | 802 (85.9) | 933 |
| **Do you have frequent headaches or neck aches?** | 195 (20.9) | 738 (79.1) | 933 |
| Do you have frequent toothaches? | 74 (7.9) | 859 (92.0) | 933 |
| Have you had a recent injury in your head, neck, or jaws? | 55 (5.9) | 877 (93.9) | 934 |
| Have you been aware of any recent changes in your bite? | 49 (5.5) | 883 (94.5) | 934 |
| Have you been previously treated for unexplained facial pain or a jaw joint problem? | 19 (2.2) | 913 (97.8) | 934 |

AAOP American Academy of Orofacial Pain; TMD temporomandibular disease
